# Operator-Managed Sedation for Pediatric Cardiac Catheterization: Experience and Safety in a Single-Center Cohort

**DOI:** 10.3390/jcm15135083

**Published:** 2026-06-30

**Authors:** Gwang-Jun Choi, Shinhyeung Kwak, Jinyoung Song, Yerin Bae, Ja-Kyoung Yoon, June Huh, I-Seok Kang

**Affiliations:** 1 Department of Pediatrics, Samsung Medical Center, Heart Vascular Stroke Institute, Sungkyunkwan University School of Medicine, Seoul 06351, Republic of Korea; okcomputer3@naver.com (G.-J.C.); irehnaeng@gmail.com (S.K.);; 2 Sungkyunkwan University School of Medicine, Suwon 16419, Republic of Korea

**Keywords:** conscious sedation, cardiac catheterization, heart defects, congenital heart disease

## Abstract

**Background/Objectives**: While the 2024 international expert consensus acknowledges that operator-managed sedation (OMS) may provide equal safety to anesthesiologist-directed care, it emphasizes clinical judgment over numerical risk scores alone, yet supporting institutional data remain limited. We evaluated the safety outcomes and risk factors for adverse events (AEs) during OMS at a single tertiary center. **Methods**: We retrospectively reviewed 342 cardiac catheterization procedures in pediatric patients under six years of age performed under OMS between 2020 and 2022. **Results**: A total of 342 cardiac catheterization procedures were performed in 307 patients, of which 30.4% were diagnostic. The overall incidence of AEs was 7.0%, and all events were successfully managed with complete recovery. In the univariate analysis, neonatal age (OR = 13.5, *p* < 0.001), body weight ≤ 10 kg (OR = 3.04, *p* = 0.01), and genetic abnormalities (OR = 3.92, *p* = 0.023) were identified as significant risk factors. Neonatal age and genetic abnormalities remained significant in the multivariate analysis. The incidence of AEs increased with higher Catheterization Risk Score for Pediatrics (CRISP) scores, showing a significant linear trend (*p* < 0.001), whereas no sedation-related AEs occurred in patients with scores below 2. Conversion to GA occurred only in patients with CRISP scores above 5. **Conclusions**: OMS can be performed safely across a wide CRISP score range in experienced centers. Neonatal age and genetic abnormalities were associated with increased AE risk; however, case selection should be guided by each patient’s overall clinical picture rather than any single risk factor alone.

## 1. Introduction

As the field of pediatric cardiac catheterization has expanded and evolved from a purely diagnostic tool into a more complex interventional procedure, the role of the anesthesiologist and general anesthesia (GA) has become increasingly important in safely maintaining hemodynamic stability and ensuring patient immobility throughout the procedure [1]. However, the routine involvement of an anesthesiologist—particularly a dedicated pediatric cardiac anesthesiologist—may be demanding in terms of personnel and institutional resources, as such specialists remain scarce even in large centers in developed countries [2]. In addition, procedures performed under sedation tend to have shorter procedure times and allow for more accurate hemodynamic data acquisition under spontaneous respiration compared to GA [3,4]. A few studies suggest that, in experienced centers, operator-managed sedation (OMS)—in which the operator supervises the sedation protocol during the procedure—may be a safe and efficient alternative to GA [3,4].

The 2016 SCAI/CCAS/SPA expert consensus statement recommended OMS only for cases with a Catheterization Risk Score for Pediatrics (CRISP) score < 2 [5]. The CRISP score was developed to predict serious adverse events (AEs) during pediatric cardiac catheterization and comprises 10 parameters—including procedure timing, age, weight, inotropic support, respiratory status, systemic illness, ASA score, physiologic category, pre-catheterization diagnosis, and procedure risk category—each assigned 0 to 2 points [6]. Accordingly, a CRISP score < 2 represents a highly limited subset of cases, and previous literature reported that only 10.3% of cases performed under OMS met this threshold [4]. The subsequently published 2024 expert consensus statement on cardiac catheterization for pediatric patients and adults with congenital heart disease (CHD) further recommends the presence of an anesthesiologist for procedures in neonates and most infants [2]. This is particularly emphasized for patients with low body weight (<4 kg), noncardiac comorbidities, or low mixed venous oxygen saturation (<50% in single ventricle physiology or <60% in biventricular circulation) [2]. However, the same consensus acknowledged that OMS “does not necessarily mean that sedation is provided with a lesser safety margin,” and explicitly stated that “clinical case-by-case judgment is equally, if not more, important” than a numerical risk score alone when selecting cases for OMS [2]. Real-world data characterizing the safety profile of OMS across a broad CRISP score range to support such judgment remain limited.

## 2. Materials and Methods

This retrospective study was conducted at a single tertiary center, Samsung Medical Center, in the Republic of Korea between January 2020 and December 2022. The study was conducted in accordance with the Declaration of Helsinki and approved by the Institutional Review Board of Samsung Medical Center (protocol code SMC 2025-11-059-001; approved on 18 November 2025). Informed consent was waived due to the retrospective study design. We reviewed pediatric cardiac catheterization cases in patients aged under six years. Patients who were mechanically ventilated, supported with non-invasive respiratory devices, or receiving oxygen therapy prior to the procedure were excluded from the analysis. Data were collected on patient age, body weight, sex, diagnosis of CHD, procedure type, comorbidities, sedative medications, procedure time, AEs, their management, and outcomes. CRISP score was calculated for each patient [6]. AEs were defined as desaturation, bradycardia, or both requiring intervention. Desaturation was defined as an oxygen saturation below 90% in patients with normal baseline saturation, or an absolute decrease of at least 10 percentage points from baseline in patients with baseline cyanosis (e.g., functional single ventricle or other cyanotic CHDs). Only events requiring intervention were recorded as AEs. AEs were further graded as minor when managed with supplemental oxygen alone or clinically significant when bag-mask ventilation, atropine administration, or endotracheal intubation was required.

### 2.1. OMS Protocol at Our Institute

During the procedure, patients were continuously monitored with pulse oximetry and electrocardiography. The operating physician determined the choice of sedative agents, as well as their doses and injection intervals, according to the patient’s condition. Sedation was primarily achieved using midazolam and ketamine, with propofol administered in selected cases. Ketamine was avoided or administered at a reduced dose in patients with respiratory infections or in those with airway or lung disease with increased secretions. Sedatives were given at either the usual dose or half the usual dose: midazolam at 0.05 or 0.1 mg/kg per dose, ketamine at 0.5 or 1 mg/kg per dose, and propofol at 0.5 or 1 mg/kg per dose or by continuous infusion. Typically, an initial dose of midazolam (0.1 mg/kg) was administered, followed by ketamine (1 mg/kg) as the second agent if additional sedation was required. A third dose of midazolam (0.05 or 0.1 mg/kg) could be given as needed, depending on the patient’s response and procedural circumstances.

Elective endotracheal intubation prior to the procedure was typically performed in neonates undergoing high-risk interventional procedures, such as balloon valvuloplasty for pulmonary atresia with intact ventricular septum, transcatheter closure (TCC) of patent ductus arteriosus (PDA) in preterm infants, right ventricular outflow tract or ductal stenting in desaturated neonates, or in patients with evidence of significant upper airway stenosis.

### 2.2. Statistical Analysis

The data were analyzed using SPSS Statistics 27.0 (IBM Corp., Armonk, NY, USA). The demographic data were presented as the number, percentage, and mean ± standard deviation (range). Categorical variables were analyzed using the chi-square test or Fisher’s exact test, as appropriate, and continuous variables were compared using the *t*-test or the Mann–Whitney U test. Multivariate analysis was performed using a binary logistic regression model to identify independent predictors of adverse events. To evaluate the trend in the incidence of adverse events across increasing CRISP scores, the linear-by-linear association test in the chi-square analysis was applied.

## 3. Results

A total of 342 cardiac catheterization procedures were performed in 307 patients younger than six years of age during the study period. A total of 169 cases were male patients (49.4%). The mean body weight was 11.5 ± 4.67 kg (range, 1.8–27.0 kg), and the mean age was 2.2 ± 1.69 years (range, 0–6 years). Among the patients, the most frequent diagnosis was PDA (20.5%), followed by atrial septal defect (15.2%), functional single ventricle (9.6%), tetralogy of Fallot (7.3%), and transposition of the great arteries (7.0%) (Table 1). Genetic abnormalities were identified in 25 patients (7.3%), including Down syndrome (6/25, 24.0%), CATCH22 syndrome (6/25, 24.0%), Williams syndrome (4/25, 16.0%), other chromosomal abnormalities (4/25, 16.0%), Alagille syndrome (3/25, 12.0%), Noonan syndrome (1/25, 4.0%), and Wolf–Hirschhorn syndrome (1/25, 4.0%).

Performed procedures are summarized in Table 2. The most frequent procedure was diagnostic catheterization, followed by TCC of PDA (19.6%), TCC of atrial septal defect (14.6%), and preoperative cardiac catheterization for Fontan or bidirectional cavopulmonary shunt surgery (9.9%). When including preoperative Fontan or bidirectional cavopulmonary shunt cases in which collateral embolization was not required, a total of 104 cases (30.4%) were diagnostic catheterizations, while 238 cases (69.6%) were interventional procedures. Among the 238 interventional procedures, the procedural success rate was 98.3% (234/238). The four unsuccessful procedures included one case of failed vascular access, two cases of failed closure for PDA and ventricular septal defect, and one case of failed sedation induction, which was successfully completed the following day under OMS.

Out of the 342 cases, 24 (7.0%) developed desaturation, bradycardia, or both requiring intervention with supplemental oxygen, bag-mask ventilation, or endotracheal intubation. Of the 24 AEs, four cases were suspected to be primarily procedure-related: three due to hemoptysis and one case of bradycardia following ventriculography with an unclear cause. When graded by clinical severity, 16 of the 24 events (66.7%) were minor and managed with supplemental oxygen alone, whereas 8 (33.3%) were clinically significant, requiring bag-mask ventilation, atropine administration, or endotracheal intubation. Endotracheal intubation was required in only 2 cases (0.6% of all procedures). The first was a 15-day-old neonate (3.2 kg) with a functional single ventricle undergoing atrial septostomy and PDA stenting (CRISP score 6, no genetic abnormality), who developed desaturation that progressed to bradycardia despite supplemental oxygen and required bag-mask ventilation followed by intubation. The second was an 8-month-old infant (6.6 kg) with Alagille syndrome and branch pulmonary artery stenosis—previously treated with balloon valvuloplasty, surgical pulmonary valvuloplasty, and bilateral pulmonary artery angioplasty—undergoing right pulmonary artery balloon angioplasty (CRISP score 7), who developed desaturation and apnea despite supplemental oxygen and required bag-mask ventilation followed by intubation. All adverse events were successfully managed, and all patients achieved complete recovery. AEs related to sedation are summarized in Table 3.

We compared demographic and clinical characteristics between patients with and without AEs (Table 4). Patients in the AE group were significantly younger and had lower body weight compared with those without AEs. Genetic abnormalities were significantly more frequent in the AE group (20.8% vs. 6.3%, *p* = 0.023). Procedure time was longer in patients with AEs (86.6 ± 44.2 vs. 65.2 ± 29.9 minutes, *p* = 0.032). Airway or lung disease was more frequent in the AE group without reaching statistical significance and anatomic complexity of CHD also tended to be higher in the AE group but was not statistically significant. In contrast, there were no significant differences between the groups regarding gender distribution, procedure type, or the use and dosing of sedative medications.

We performed a risk factor analysis for AEs occurring during OMS (Table 5). In the univariate analysis, neonates had 13.5-fold higher odds of AEs compared with preschool children (OR = 13.5, 95% CI 3.32–54.9, *p* < 0.001). Patients weighing ≤10 kg also showed a significantly higher risk (OR = 3.04, 95% CI 1.26–7.31, *p* = 0.01). Genetic abnormalities were associated with increased risk (OR = 3.92, 95% CI 1.32–11.6, *p* = 0.023). Other variables, including sex, procedure type (diagnostic versus interventional), airway disease, prematurity, and propofol usage, were not significantly associated with AEs. In multivariate analysis, neonatal age (OR = 8.94, 95% CI: 2.11–37.86, *p* = 0.003) and genetic abnormalities (OR = 3.94, 95% CI: 1.25–12.39, *p* = 0.019) were independently associated with the occurrence of AEs under OMS.

Stratification by CRISP score revealed an increasing incidence of AEs with higher scores (Table 6). No AEs were observed in patients with scores < 2. The incidence rose modestly to 6.0% at a score of 2, 8.1% at 3, and 5.7% at 4, and then increased substantially to 17.1% at 5, 30.8% at 6, and 50.0% at 7. A significant linear trend was demonstrated between CRISP score and AE incidence by the linear-by-linear association test (*p* < 0.001). In patients with a CRISP score of 2, only a single event necessitating bag-mask ventilation was observed, which was attributed to bradycardia following ventriculography. The association with sedation was unclear. Two patients required endotracheal intubation during the procedure, and their CRISP scores were 6 and 7, respectively.

## 4. Discussion

We investigated the incidence of AEs during pediatric cardiac catheterization performed under OMS. The overall frequency of AEs was 7.0%, and all affected patients were managed appropriately and recovered completely. Neonatal age and genetic abnormalities were independent risk factors for adverse events. The CRISP score showed a strong correlation with the incidence of adverse events under OMS. Notably, no sedation-related AEs occurred in patients with a CRISP score below 2.

The overall rate of AEs was 7.0%, and the rate of conversion to GA was low (0.6%). AEs during OMS have been reported to occur in 2.2–9% of cases in the literature, and the incidence of AEs in our study was within this range, even though the majority of procedures were performed under OMS. Younger age, smaller body size, lower mixed venous oxygen saturation, and the presence of comorbidities have been identified as risk factors for AEs in the literature [7,8]. In this study, neonatal age, smaller body weight (<10 kg), and the presence of genetic abnormalities were significant in univariate analysis. In the multivariate analysis, neonatal age and genetic abnormalities remained significant independent risk factors.

Of note, genetic abnormality emerged as an independent predictor of AEs in our cohort (OR 3.94, *p* = 0.019), a finding that has not been previously highlighted in the context of OMS case selection [2]. Genetic abnormalities are associated with a range of features that may predispose to sedation-related AEs, including upper airway anomalies, hypotonia, and heightened sensitivity to sedative agents. However, this category is clinically heterogeneous: among the patients with a genetic abnormality who experienced an AE, the underlying conditions included Down syndrome, which is prone to upper airway problems; CATCH22 syndrome, which is frequently associated with velopharyngeal insufficiency and airway malacia; and Alagille syndrome, in which branch pulmonary artery stenosis often necessitates painful balloon angioplasty and thus deeper sedation. This association may therefore reflect these underlying features rather than the genetic diagnosis itself, and residual confounding within this heterogeneous subgroup cannot be excluded. Accordingly, because such features are not captured by the CRISP score, the presence of a genetic abnormality should inform individualized, case-by-case judgment during OMS case selection rather than being treated as a fixed independent risk factor.

We used midazolam, ketamine, and/or propofol for sedation, and there were no significant differences between the AE and non-AE groups in either the drug doses or the use of propofol. The sequence of agent administration (e.g., midazolam alone, ketamine alone, or sequential midazolam–ketamine–midazolam) was likewise not associated with the occurrence of AEs (*p* = 0.21). In our practice, additional doses and agents were titrated in real time according to each patient’s depth of sedation and procedural circumstances, which makes the regimen difficult to standardize into fixed protocols. For this reason, and given the limited number of events, a robust analysis of specific drug combinations was not feasible; nonetheless, no particular regimen emerged as being associated with AEs in our cohort. A few recent studies have suggested the use of dexmedetomidine as an adjunctive sedative because it is associated with less frequent oxygen supplementation and a reduced requirement for midazolam [9,10]. Further studies are needed to determine the optimal sedative regimen for OMS in pediatric cardiac catheterization.

As pediatric cardiac catheterization has become more complex and interventional, the role of specialized anesthetic care has been increasingly emphasized [1]. According to the 2024 expert consensus statement, no updated guidelines regarding anesthetic care for pediatric cardiac catheterization have been published since 2016 [2,5]. The guideline recommended the involvement of specialized anesthesiologists for all procedures except those with a CRISP score of 0 or 1 [5]. Data from a U.S. multicenter registry showed that OMS was used infrequently (19% in 2011 and 3.9% in 2018) [3]. OMS was performed safely, without an increased incidence of major AEs, and was associated with reduced fluoroscopy and total procedure times [3]. The study also revealed that only 12% of patients who received OMS were considered appropriate according to the 2016 guideline recommendations [3]. In this study, most procedures were performed under OMS, and only 19 cases (5.3%) were conducted after elective intubation during the study period. The majority of patients (71%) had a CRISP score of 2 or greater. Airway events were almost absent in patients with a CRISP score ≤ 2 (with one unrelated event in a CRISP 2 case), while conversions to GA during the procedure were observed only in patients with a CRISP score > 5. These findings may suggest that the CRISP score cut-off of <2 proposed in the previous guideline was conservative. However, the successful management of unexpected AEs during OMS likely depends on the experience and expertise of the institutional team, and our single-center data cannot define a generalizable threshold; a less stringent CRISP cut-off may not be universally appropriate [2].

This study has several limitations. First, it was a single-center study with a limited number of patients; therefore, its results may not be widely generalizable. Second, our analysis may have been underpowered because of the low incidence of AEs and the relatively small sample size. Given the limited number of events relative to the variables examined, the independent predictors identified in the multivariable analysis require further validation. In addition, no parallel cohort undergoing anesthesiologist-directed care or general anesthesia was available for comparison. Our data therefore describe the safety profile of OMS within a single practice model but cannot establish the equivalence or superiority of OMS, and the favorable outcomes observed across a broad CRISP range should be interpreted accordingly. Third, the sedation protocol and the operator-based airway-management model used at our center reflect local practice patterns, training, and regulatory context. The administration of propofol, ketamine, and midazolam under the direction of a non-anesthesiologist operator, as well as airway rescue—including endotracheal intubation—performed by the cardiology team, would be considered unconventional in many countries, where many cardiologists neither expect nor are equipped to perform tracheal intubation. Our findings should therefore be interpreted in the context of an experienced team with such capabilities and may not be generalizable to institutions with different settings. Hence, future studies with larger, multicenter cohorts are warranted to confirm our findings.

## 5. Conclusions

Pediatric cardiac catheterization can be performed safely under OMS in the majority of cases, with a low overall incidence of AEs, all of which were successfully managed in our experienced center. The strong correlation between CRISP score and AE incidence supports its utility as a risk stratification tool. Neonatal age was a strong independent predictor of AEs. Genetic abnormalities were also associated with increased AE risk; however, given the heterogeneity of this category, case selection should be guided by each patient’s overall clinical picture rather than by the genetic diagnosis alone.

## Figures and Tables

**Table 1:** Diagnosis for Cardiac Catheterization under Operator-Managed Sedation.

Diagnosis	Cases (%)
Patent ductus arteriosus	70 (20.5%)
Atrial septal defect	52 (15.2%)
Functional single ventricle	33 (9.6%)
TOF/TOF-type DORV	25 (7.3%)
TGA/TGA-type DORV	24 (7.0%)
Pulmonary stenosis	21 (6.1%)
Others	21 (6.1%)
Ventricular septal defect	20 (5.8%)
Branch pulmonary artery stenosis	19 (5.6%)
PA VSD	14 (4.1%)
CoA/IAA/AS	12 (3.5%)
Kawasaki disease	8 (2.3%)
Pulmonary hypertension	6 (1.8%)
PA IVS	6 (1.8%)
Coronary artery fistula	5 (1.5%)
Pericardial effusion	3 (0.9%)
Atrial septal defect with pulmonary stenosis	2 (0.6%)
Ventricular septal defect with pulmonary stenosis	1 (0.3%)
Total	342 (100.0%)

Abbreviations: TGA, transposition of the great arteries; DORV, double outlet right ventricle; PA VSD, pulmonary atresia with ventricular septal defect; TOF, tetralogy of Fallot; PA IVS, pulmonary atresia with intact ventricular septum; CoA, coarctation of the aorta; IAA, interrupted aortic arch; AS, aortic stenosis.

**Table 2:** Cardiac catheterization procedures.

Procedure	Frequency (%)
Diagnostic catheterization	86 (25.1)
PDA device closure	67 (19.6)
ASD device closure	50 (14.6)
Pre-Fontan/BCPS diagnostic study with/without collateral embolization	34 (9.9)
Pulmonary balloon valvuloplasty	33 (9.6)
Branch PA balloon angioplasty	25 (7.3)
VSD device closure	17 (5.0)
Branch PA stent placement	7 (2.0)
Pericardiocentesis	5 (1.5)
Collateral embolization	4 (1.2)
PDA stenting	3 (0.9)
Heart biopsy	3 (0.9)
Coronary artery fistula closure	2 (0.6)
Atrial septostomy	2 (0.6)
Aortic balloon valvuloplasty/residual coarctation angioplasty	2 (0.6)
TPM insertion	1 (0.3)
ASD device closure and Pulmonary BVP (combined procedure)	1 (0.3)

Abbreviations: ASD, atrial septal defect; PDA, patent ductus arteriosus; PA, pulmonary artery; BCPS, bidirectional cavopulmonary shunt; VSD, ventricular septal defect; TPM, temporary pacemaker.

**Table 3:** Adverse events related to sedation and their management.

Adverse Events	Number (%)
Desaturation	20 (83)
Desaturation and apnea/bradycardia	3 (13)
Bradycardia	1 (4)
**Management**	
Oxygen mask	16 (67)
Bag-mask ventilation	5 (21)
Bag-mask ventilation and atropine administration	1 (4)
Intubation	2 (8)
**Recovery**	
Complete recovery	24 (100)

**Table 4:** Comparison of demographics and clinical characteristics between adverse event and no-event groups.

Demographic and Clinical Characteristics	No AE	AE	*p*-Value
**Gender, Male (%)**	158 (49.7)	11 (45.8)	0.716
**Average age, years**	2.28 ± 1.68	1.63 ± 1.83	0.072
**Average weight, kg**	11.75 ± 4.59	8.99 ± 5.21	<0.001
**Anatomic complexity of CHD**			0.088
-Simple lesion or normal structure	192 (95.5)	9 (4.5)	
-Complex CHD with biventricular heart	95 (89.6)	11 (10.4)	
-Complex CHD with FSV	31 (88.6)	4 (11.4)	
**Born preterm**	36 (11.3)	2 (8.3)	1.000
**Airway/lung disease**	12 (3.8)	3 (12.5)	0.079
**Genetic abnormalities**	20 (6.3)	5 (20.8)	0.023
**Procedure (%)**			0.747
-Diagnostic	96 (92.4)	8 (7.6)	
-Intervention	222 (93.3)	16 (6.7)	
**Procedure time (min)**	65.21 ± 29.88	86.57 ± 44.20	0.032
**Dose of midazolam, mg/kg/h**	0.33 ± 0.30	0.36 ± 0.19	0.633
**Dose of ketamine, mg/kg/h**	2.27 ± 1.46	1.80 ± 1.02	0.120
**Use of propofol**	35 (11.0)	3 (13.0)	0.731

Abbreviations: AEs, adverse events; CHD, congenital heart disease; FSV, functional single ventricle.

**Table 5:** Risk factors for sedation-related adverse events.

Variables	Univariate OR (95% CI)	*p*-Value	Multivariate OR (95% CI)	*p*-Value
**Sex, male**	1.17 (0.51–2.68)	0.716		
**Age group**				
1–6 years (ref)				
Neonate	13.5 (3.32–54.94)	<0.001	8.94 (2.11–37.86)	0.003
Infant	2.05 (0.82–5.12)	0.124		
**Procedure**				
Diagnostic (ref)				
Intervention	0.87 (0.36–2.09)	0.747		
**Bodyweight**				
>10 kg (ref)				
≤10 kg	3.04 (1.26–7.31)	0.010	1.96 (0.75–5.11)	0.170
**Underlying disease**				
Airway/lung disease	3.64 (0.95–13.92)	0.079 ^a^		
Genetic abnormalities	3.92 (1.32–11.60)	0.023 ^a^	3.94 (1.25–12.39)	0.019
Born preterm	0.71 (0.16–3.16)	1.000 ^a^		
**Propofol usage**	1.21 (0.34–4.29)	0.731 ^a^		

Abbreviations: CI, confidence interval, ^a^ Fisher’s exact test.

**Table 6:** Incidence of sedation-related adverse events according to the CRISP score.

CRISP Score	Total	AEs (%)	Bag-Mask Ventilation	Intubation
0	27	0 (0)	0	0
1	71	0 (0)	0	0
2	67	4 (6.0)	1	0
3	74	6 (8.1)	3	0
4	53	3 (5.7)	0	0
5	35	6 (17.1)	0	0
6	13	4 (30.8)	2	1
7	2	1 (50.0)	0	1
Total	342	24	6	2

Abbreviations: AE, adverse events.

## Data Availability

The datasets generated or analyzed during the current study are available from the corresponding author on reasonable request.
